# Study on the Disinfection Efficacy of Common Commercial Disinfectants in China Against Mastitis-Causing Pathogens and Bedding Materials in Large-Scale Dairy Farms

**DOI:** 10.3390/vetsci12111072

**Published:** 2025-11-08

**Authors:** Tianchen Wang, Haoyu Fan, Mengqi Chai, Tao He, Yongqi Li, Xiangshu Han, Yanyang Li, Hangfei Bai, Song Jiang

**Affiliations:** 1 College of Animal Sciences, Shihezi University, Shihezi 832000, China; 18083933115@163.com (T.W.); fanhaoyu@xjshzu.com (H.F.); cmqtgzy@163.com (M.C.); ht960704@163.com (T.H.); ybwrsys@163.com (X.H.); 18865345100@163.com (Y.L.); xiaobailajia123@163.com (H.B.); 2 College of Animal Science and Technology, Southwest University, Chongqing 402460, China; 14719869471@163.com

**Keywords:** livestock farms, disinfectants, mastitis, influencing factors, cubicle bedding

## Abstract

**Simple Summary:**

To address the issue of mastitis in large-scale dairy farms caused by *Escherichia coli* and *Staphylococcus aureus*, as well as the problems of traditional disinfection methods relying on experience and having unstable efficacy, this study aimed to identify optimal disinfection strategies suitable for different scenarios and seasons. Five common disinfectants were tested under conditions including bacterial suspension, stainless steel carriers (simulating milking equipment), cow dung bedding, and different temperatures. Results showed that the glutaraldehyde compound preparation exhibited the best and most stable efficacy; its combination with calcium hypochlorite achieved the optimal effect in controlling bacterial rebound in cow dung bedding. This study clarified the scenario-specific adaptation rules of disinfectants and established a precise scenario-based disinfection strategy, which can improve mastitis prevention and control, optimize biosafety, and also provide references for disinfection in fields such as medical care and food processing.

**Abstract:**

To address the challenges in preventing and controlling mastitis caused by *Escherichia coli* and *Staphylococcus aureus* in large-scale dairy farms, as well as the issues of traditional disinfection protocols relying on experience and exhibiting significant efficacy fluctuations, this study aimed to systematically explore optimal disinfection strategies adapted to different scenarios and seasons. Five common commercial disinfectants in China were selected to target the two aforementioned pathogenic strains. Experiments were conducted under three typical scenarios—bacterial suspension, stainless steel carriers (simulating milking equipment), and cow dung cubicle bedding—and three temperature conditions (4 °C, 25 °C, 37 °C, simulating seasonal temperatures). A series of tests were performed, including neutralizer identification tests, determination of minimum inhibitory concentration (MIC) and minimum bactericidal concentration (MBC), quantitative suspension and carrier spray disinfection tests, and monitoring of bacterial growth and decline in cow dung cubicle bedding. These tests were used to quantitatively analyze the regulatory mechanisms of disinfectant concentration, action time, and environmental temperature on disinfection efficacy. The Compound Glutaral Solution (CGS) exhibited the best overall performance, with strong temperature stability across all scenarios and high-efficiency bactericidal activity even at low concentrations. Additionally, the combined system of the CGS and bleaching powder (BP) achieved the optimal effect in controlling bacterial rebound in the cow dung cubicle bedding scenario. This study clarified the scenario-specific adaptation rules of different disinfectants and established a scenario-specific precision disinfection strategy for dairy farms. It provides a scientific basis for improving the level of mastitis prevention and control and optimizing biosafety systems, while also offering references for the disinfection of hard surfaces in fields such as healthcare and food processing.

## 1. Introduction

In the global transition of animal husbandry toward intensification and large-scale operations, high-density farming models have significantly enhanced the transmission efficiency of pathogens while accelerating the spread of drug-resistant microorganisms, posing severe challenges to the prevention and control of animal diseases [1]. Among these diseases, bovine mastitis stands as the most economically damaging contagious disease in the global dairy industry, with its incidence showing a significant positive correlation with the imbalance of the microecology in the farming environment [2]. This disease not only reduces milk yield and degrades milk quality but also exacerbates the pressure of breeding costs due to increased dairy cow culling rates. Notably, its core transmission pathways are directly linked to the hygiene status of milking equipment and the contamination of cubicle bedding [3]. The continuous contact between dairy cow udders and contaminated bedding serves as a major trigger for pathogen colonization. Among the dominant pathogens causing clinical and subclinical mastitis, *E. coli* and *S. aureus* account for over 65% [4]; these two pathogens can form dense biofilms on the surface of bedding substrates, encapsulating bacterial cells within an extracellular polysaccharide matrix. This structure significantly prolongs their survival time, enhances their stress resistance, and increases the risk of cross-infection [5]. Statistics indicate that the global direct economic loss caused by bovine mastitis exceeds 35 billion US dollars annually [6]. Meanwhile, issues such as elevated somatic cell counts [7] and pathogenic residue [8] in raw milk pose potential threats to public health.

The traditional mastitis prevention and control system relies heavily on the prophylactic use of antibiotics, which are administered before/after milking or during the dry period to inhibit pathogen colonization [9]. However, following the implementation of China’s comprehensive ban on antibiotics in feed in 2020, the application of antibiotics in the breeding sector has been strictly restricted. The livestock industry thus needs to break free from “antibiotic dependence” and establish a novel prevention and control system centered on biosafety. Disinfection, as a key link in interrupting the environmental transmission chain of pathogens, directly determines the efficiency of mastitis prevention and control. By reducing the pathogen load in critical scenarios such as cubicle bedding and milking equipment, it can minimize the risk of udder contact infection at the source [10], playing an irreplaceable role in reducing antibiotic use and improving animal welfare.

Despite the wide variety of disinfectants available on the market (e.g., iodine-containing, quaternary ammonium salt, and aldehyde-based products), the practical disinfection efficacy in large-scale dairy farms often exhibits significant fluctuations due to interference from multiple factors. The type of disinfectant determines its core mechanism of action [11]; concentration and action time affect the efficiency of effective contact [12]; and environmental temperature (e.g., seasonal temperature variations in dairy farms, ranging from 4 to 37 °C) further influences bactericidal efficacy by regulating the metabolic activity of pathogens and the molecular movement rate of disinfectants [13]. Currently, most farms still select disinfectants based on experience [14], lacking scientific verification targeting the core pathogens of dairy farms and typical scenarios (high-organic-matter environments of cubicle bedding, hard surfaces of milking equipment). This prevalence of empirical selection leads to common problems such as “high cost with low efficacy” and “poor seasonal adaptability.”

Guided by the prevention and control needs of environmental mastitis [15] (i.e., mastitis in dairy cows caused by pathogenic bacteria in the environment) in large-scale dairy farms, this study focuses on two dominant pathogenic bacteria in the environment, *E. coli* and *S. aureus*. Given that improper disinfection practices in actual production (e.g., failure to dilute disinfectants according to the recommended ratio) may induce bacterial resistance to disinfectants, we first determine the minimum inhibitory concentration (MIC) and minimum bactericidal concentration (MBC) of disinfectants against the two pathogenic bacteria to clarify their actual disinfection range and effective action thresholds. On this basis, combined with the actual application scenarios of dairy farms (manure in cubicles, surfaces of stainless steel equipment) and seasonal temperature differences (4 °C, 25 °C, 37 °C), we systematically explore the regulatory mechanisms of disinfectant type, concentration, and action time on disinfection efficacy. By quantitatively evaluating the killing rate and persistence of different disinfection schemes, we ultimately identify the optimal disinfection strategies adapted to different scenarios and seasons in dairy farms, providing a solid scientific basis for optimizing the biosafety system and improving the level of mastitis prevention and control in large-scale dairy farms.

## 2. Materials and Methods

### 2.1. Materials

Test Disinfectants: Polyvinylpyrrolidone–Iodine Solution (PVP-I): Qilu Animal Health Products Co., Ltd., Jinan, China (Specification: 5%). Potassium Peroxymonosulfate Complex Powder (PMBC): Chengdu Minsheng Disinfectant Co., Ltd., Chengdu, China (Specification: available chlorine content of no less than 10%).

Glutaraldehyde–Didecyldimethylammonium Bromide Solution (G-DDB): Sichuan Dingjian Animal Pharmaceutical Co., Ltd., Chengdu, China (Specification: 100 mL contains 5 g glutaraldehyde + 5 g didecyldimethylammonium bromide). Compound Glutaraldehyde Preparation (CGS): Coventry Chemicals Co., Ltd., Coventry, UK (Specification: 100 mL contains 15 g glutaraldehyde + 10 g benzalkonium chloride). Benzalkonium Bromide Preparation (BKB): Shijiazhuang Shimuyang Pharmaceutical Co., Ltd., Shijiazhuang, China (Specification: 5%).

Bacterial Strains: *E. coli* and *S. aureus* were both isolated and preserved from dairy farm environments by the Laboratory of Clinical Veterinary Medicine, Shihezi University.

### 2.2. Preparation of Bacterial Suspension

Glycerol-preserved strains stored at −20 °C were taken, and 10.0 mL of LB medium was added to 50 mL test tubes. 100 μL of the bacterial suspension was inoculated at a 1:100 ratio, followed by incubation at 37 °C overnight. The colony concentration was then determined using the plate count method. The strains were streak-inoculated onto solid LB medium slants and cultured at 37 °C for 16 h. Subsequently, typical colonies were picked for enrichment culture in LB medium. According to the concentration corresponding to the plate dilution method, the bacterial suspension was adjusted and diluted to 1 × 10^8^–3 × 10^8^ CFU/mL.

### 2.3. Neutralizer Identification Test

This test was designed in accordance with the Standards for Disinfection Technology [16]. Suitable neutralizers (see Table 1 for details) were selected to establish the evaluation system. First, 2 mL of the test bacterial suspension was premixed with an equal volume of 6% bovine serum albumin (BSA) in a constant-temperature water bath at 20 ± 1 °C. Thereafter, the mixture was treated according to the following experimental groups:

Group 1 (Disinfectant Action Group): 1 mL of the bacterial suspension was quantitatively pipetted and equilibrated in a 24 °C water bath for 5 min. Then, 4 mL of the target disinfectant (PVP-I, PMBC, G-DDB, CGS, or BKB) was added and mixed uniformly. Immediately afterward, a 0.5 mL aliquot was sampled, diluted at a ratio of 1:10, and 1 mL of the diluted sample was inoculated for culture to determine the baseline colony count.

Group 2 (Neutralizer Verification Group): On the basis of the treatment in Group I, an additional 2 mL of BSA was added. After a 10 min reaction, serial dilution and culture for colony counting were performed.

Group 3 (Positive Control Group): 0.1 mL of the original bacterial suspension was diluted with sterile hard water and directly cultured to verify the initial bacterial concentration (5 × 10^5^–5 × 10^6^ CFU/mL).

Group 4 (Neutralization Product Group): The bacterial suspension was reacted with 4.9 mL of the neutralization product solution for 10 min, followed by culture to evaluate the interference of the neutralizer.

Group 5 (Diluent Control Group): The bacterial suspension was replaced with diluent to verify the sterility of the matrix.

Group 6 (Medium Verification Group): Diluent, neutralizer, and medium were mixed and cultured to confirm that no reagent contamination occurred.

#### Evaluation Criteria

The colony count of Group 1 was significantly lower than that of Group 2 (*p* < 0.05).

The bacterial concentrations of Groups 3-5 remained relatively stable (inter-group deviation rate ≤ 15%).

Absolutely no bacterial growth was allowed in Group 6; otherwise, reagent contamination was determined.

The coefficient of variation in data from 3 consecutive independent experiments was <10%.

Formula for calculating the error rate of colony count: The inter-group colony error rate is equal to the sum of the absolute values of the difference between the average number of colonies among the three groups and the average number of colonies in each group, divided by three times the average number of colonies among the three groups.

### 2.4. Determination of Minimum Inhibitory Concentration (MIC) and Minimum Bactericidal Concentration (MBC) of Disinfectants

The concentration ranges of disinfectants with inhibitory and bactericidal effects on target strains were screened out via MIC/MBC. The stock solution of each disinfectant was diluted to 4× its working concentration using sterile hard water. For a 96-well plate, 190 μL of medium was pre-added to columns A1–H1, while 100 μL of medium was dispensed into all other wells. A 10 μL aliquot of the disinfectant stock solution was injected into wells A1–H1 (initial dilution ratio: 1:5); after thorough mixing, serial gradient dilution (1:5→1:5120) was performed, with 100 μL transferred for each gradient step.

After transferring the diluted samples to a new 96-well plate, 20 μL of the gradient sample solution was added to each well (well H12 served as the blank control), followed by inoculation with 180 μL of bacterial suspension at a concentration of 1.5 × 10^5^–1.0 × 10^6^ CFU/mL (Final disinfectant dilution gradient 1:50~1:51,200). The plate was incubated at 37 °C for 16–20 h; the sterility of the blank control well was used as the criterion to validate the experiment’s effectiveness. The dilution ratio corresponding to the first well with no bacterial growth was defined as the minimum inhibitory concentration (MIC).

Following 24 h of incubation for MIC determination, the well corresponding to the MIC value and wells with higher concentration gradients (2 × MIC, 4 × MIC) were selected. After homogenizing the bacterial suspension in these wells, 100 μL of the sample was pipetted and evenly spread onto labeled agar plates. The plates were incubated in a 37 °C constant-temperature incubator for 16–18 h, and the highest dilution gradient with no colony growth was identified as the minimum bactericidal concentration (MBC).

### 2.5. Quantitative Bactericidal Test on Bacterial Suspension

Prepare five disinfectants according to the instructions, each with four concentration gradients (MBC, MIC, 1/2MIC, and 1/4MIC) for different bacterial strains. Prepare 50 mL of each gradient and incubate in a 20 ± 1 °C water bath to achieve equilibrium before use. Concurrently, the concentration of the bacterial suspension was adjusted to 1 × 10^8^–5 × 10^8^ CFU/mL to ensure the accuracy of the test bacterial concentration.

The disinfection reaction system was established as follows [16] (with reference to Laboratory Test Methods for Bactericidal Efficacy of Disinfectants): 0.5 mL of standardized bacterial suspension and 0.5 mL of 6% bovine serum albumin (used as an organic interfering substance) were sequentially added to a sterile test tube. After equilibration in a 37 °C water bath for 5 min, 4 mL of the pre-warmed disinfectant solution (equilibrated to the target temperature) was injected into the tube, followed by vortex oscillation for 30 s to achieve complete mixing.

At preset time points, 0.5 mL of the above mixture was transferred to a test tube containing 4.5 mL of the corresponding neutralizer (as specified in Table 1), and vortex oscillation was conducted for 30 s to terminate the disinfection reaction. Subsequently, 100 μL of the reaction-terminated solution was quantitatively pipetted and inoculated onto nutrient agar plates for microbial quantification. Finally, the inoculated plates were incubated at 37 °C for 24 h, and the number of viable colonies was counted; only plates with ≥30 CFU were considered valid data.

The bacterial killing rate was calculated using the formula: (Viable count in the control group−Viable count in the treatment group)/Viable count in the control group × 100%. Each experiment was independently repeated 3 times, and the final results were expressed as the mean ± standard deviation of the three replicates.

### 2.6. Quantitative Bactericidal Test for Carrier Spray Disinfection

Disinfectants were prepared according to the aforementioned method, and carrier bacterial slides were prepared in accordance with Laboratory Test Methods for Bactericidal Efficacy of Disinfectants [17]. The stainless steel carriers used were 304 stainless steel sheets (size: 2 cm × 5 cm, surface roughness Ra = 0.8 μm). Prior to use, they were autoclaved at 121 °C for 30 min, degreased by wiping with absolute ethanol, rinsed with sterile water, and then dried.

Carrier bacterial slides were placed in sterile Petri dishes using the drop-inoculation method. A pipette was used to add bacterial suspension (concentration: 1 × 10^8^ CFU/mL) dropwise to each slide; if necessary, an inoculating loop was used to spread the suspension evenly on the carrier surface. The slides were then placed in an incubator at 37 °C with 60% relative humidity to dry for 2 h, ensuring the initial bacterial load on each carrier was 1 × 10^6^–5 × 10^6^ CFU per slide.

Disinfectant was sprayed using a manual sprayer with a pressure of 0.2 MPa, a spray distance of 15 cm, and a spray volume of 0.5 mL per carrier—this ensured uniform size and quantity of spray droplets while avoiding saturation of the bacterial slides.

After the disinfection contact time, the carriers were transferred into 5 mL of neutralizer (formulation in Table 1) and oscillated for elution. A 100 μL aliquot of the eluate was spread on nutrient agar plates, which were incubated at 37 °C for 24 h for colony counting. At the preset time points, the bacterial slides were transferred into 5.0 mL of neutralizer; after mixing, 100 μL of the eluate was taken for counting.

Colony culture counting and bacterial killing rate calculation were performed according to the aforementioned methods, with results expressed as the mean ± standard deviation of three replicates per group.

### 2.7. Disinfection of Cubicle Bedding in Dairy Barns

One test area was randomly selected in a lactating dairy barn, with 120 free-access cubicles in each test area. Cow dung was used as cubicle bedding, and the cow dung was sterilized before use. Three disinfectants with good disinfection efficacy in laboratory tests (PMBC, G-DDB, and CGS) were selected. Under the barn temperature condition of 18 °C ± 2 °C, the bactericidal efficacy of the disinfectants on cow dung cubicles and barn walls was determined, and the bacterial growth and decline pattern was monitored.

Ten cubicles were randomly selected as sampling subjects in each test area. When dairy cows were taken to the milking parlor for milking, quicklime was sprinkled on the empty cubicles and mixed thoroughly, followed by spray disinfection with PMBC, G-DDB, and CGS solutions, respectively.

## 3. Results

### 3.1. Results of Neutralizer Identification Test

Use *Escherichia coli* as the reference strain. Broth medium containing 0.1% sodium thiosulfate could effectively neutralize the residual toxicity of PVP-I to the test bacteria; broth medium containing 3% Tween 80 could effectively neutralize the residual toxicity of BKB to the test bacteria; broth medium containing 3% Tween-80 + 0.5% lecithin + 0.3% glycine could effectively neutralize the residual toxicity of G-DDB and CGS to the test bacteria; and broth medium containing 2% Tween-80 + 0.1% lecithin + 0.5% sodium thiosulfate could effectively neutralize the residual toxicity of PMBC to the test bacteria. Additionally, neither the neutralizers nor their neutralization products had any effect on the growth and culture of the test bacteria (Table 2).

### 3.2. Determination Results of MIC and MBC Values of the Five Disinfectants Against Isolated Strains

Based on the microdilution method (Table 3), the bacteriostatic concentrations of the five disinfectants against *S. aureus* and *E. coli* showed significant differences. The results indicated that PVP-I exhibited initial bacteriostatic activity at a 1:16 dilution, with a minimum inhibitory concentration (MIC) of 1:8. The MIC values of PMBC and G-DDB were 1:800 and 1:960, respectively, while BKB (1:1280) and CGS (1:3840) could achieve bacteriostatic effects at relatively low concentrations.

Data in Table 4 showed that under room temperature conditions, the minimum bactericidal concentrations (MBC) of the five disinfectants against *E. coli* and *S. aureus* presented a gradient distribution: PVP-I (1:4) > BKB (1:160) > PMBC (1:400) > G-DDB (1:480) > CGS (1:640).

### 3.3. Quantitative Bactericidal Test in Suspension

#### 3.3.1. Bactericidal Efficacy of Five Disinfectants Against *E. coli*

**Figure 1:**
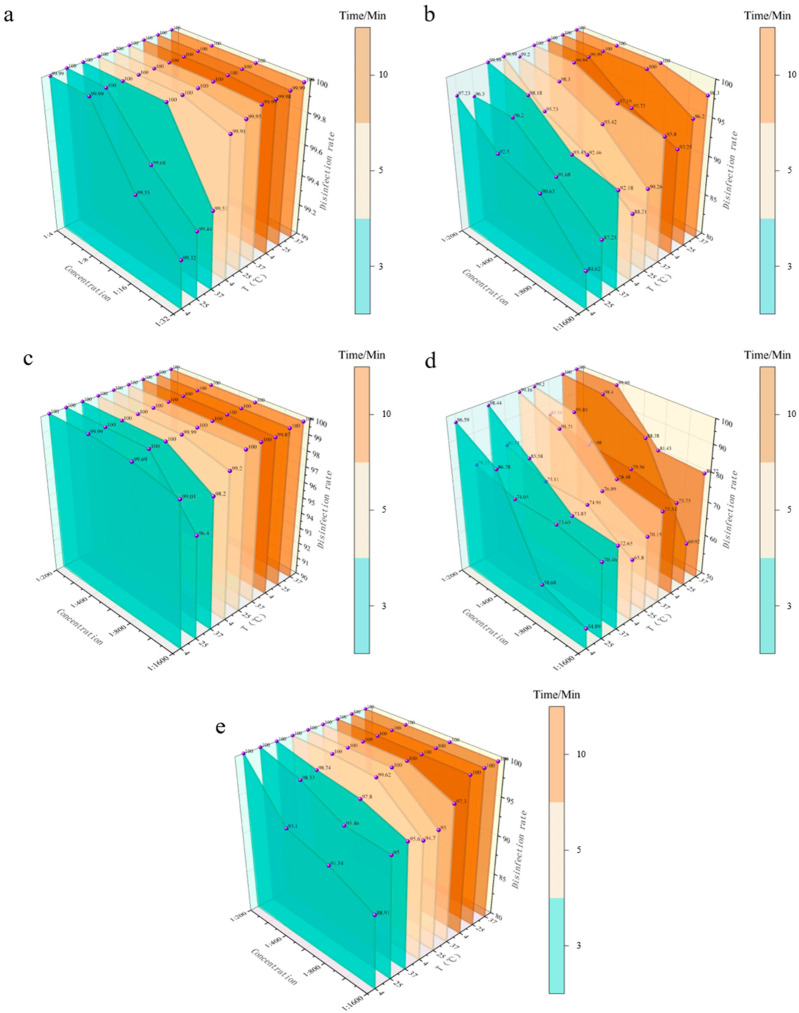
Bacterial reduction rate of disinfectants against *E. coli* in aqueous suspension. (**a**) Bactericidal efficiency of PVP-I. (**b**) Bactericidal efficiency of BKB. (**c**) Bactericidal efficiency of CGS. (**d**) Bactericidal efficiency of G-DDB. (**e**) Bactericidal efficiency of PMBC. The *X*-axis represents disinfectant concentration, the *Y*-axis represents disinfection efficiency, and the *Z*-axis represents different temperatures. Different colors represent different contact times (3, 5, 10 min).

To provide evidence-based guidance for disinfection practices, this study systematically evaluated the bactericidal efficacy of five commonly used disinfectants in clinical and industrial settings (PVP-I, BKB, CGS, G-DDB, and PMBC) against *E. coli* under idealized conditions (bacterial suspension). Quantitative bactericidal tests were conducted to obtain killing rate data, with multiple experimental variables: disinfectant concentration (four gradients per disinfectant), contact time (3, 5, 10 min), and environmental temperature (4, 25, 37 °C). These variables comprehensively revealed the effects of different conditions on disinfection efficiency (specific data in Figure 1 and Appendix Table A1).

Under all tested conditions, the bactericidal efficacy of the five disinfectants against *E. coli* was ranked as: CGS > PVP-I ≈ PMBC > BKB > G-DDB. Key observations included: CGS demonstrated unmatched temperature stability and low concentration requirements, making it the optimal choice for diverse scenarios. PVP-I and PMBC achieved a favorable balance between efficacy and adaptability: PVP-I excelled in rapid disinfection at low concentrations, while PMBC compensated for low temperatures through extended contact times. BKB required strict control of concentration and temperature to ensure effectiveness, limiting its application scope. G-DDB consistently exhibited poor performance across all conditions.

#### 3.3.2. Bactericidal Efficacy of Five Disinfectants Against *S. aureus*

**Figure 2:**
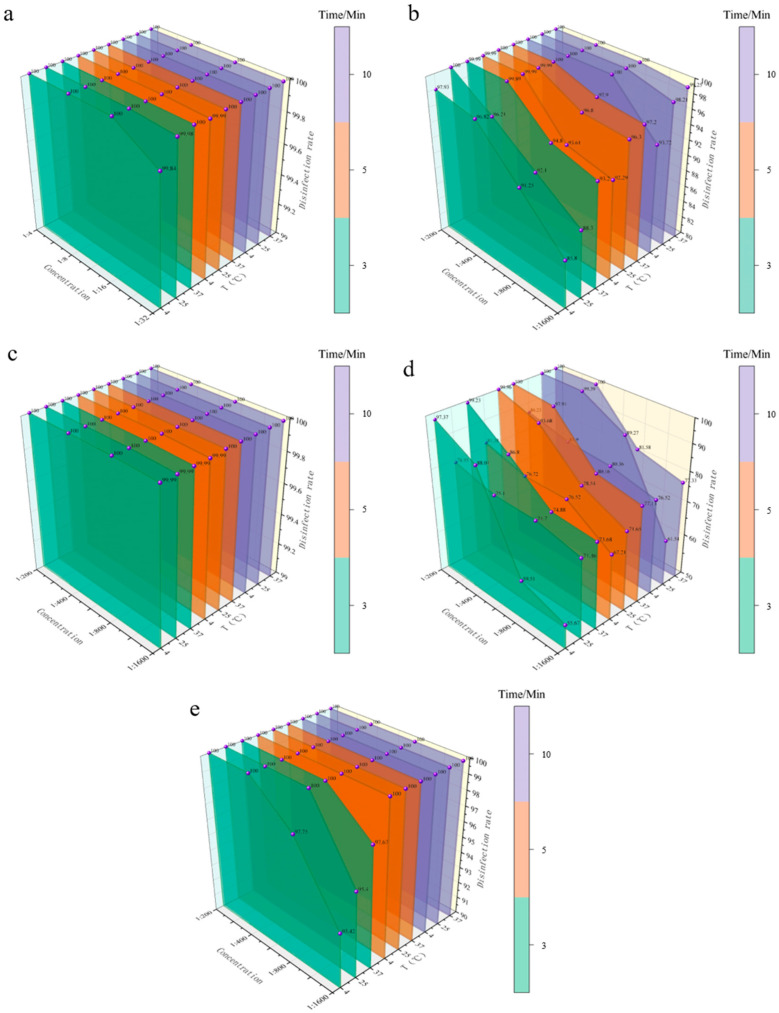
Bacterial reduction rate of disinfectants against *S. aureus* in aqueous suspension. (**a**) Bactericidal efficiency of PVP-I. (**b**) Bactericidal efficiency of BKB. (**c**) Bactericidal efficiency of CGS. (**d**) Bactericidal efficiency of G-DDB. (**e**) Bactericidal efficiency of PMBC. The *X*-axis represents disinfectant concentration, the *Y*-axis represents disinfection efficiency, and the *Z*-axis represents different temperatures. Different colors represent different contact times (3, 5, 10 min).

*S. aureus*, a common pathogenic Gram-positive bacterium in clinical and environmental settings, poses stringent challenges to disinfection protocols due to its drug resistance and survival capabilities. This study evaluated the bactericidal efficacy of PVP-I, BKB, CGS, G-DDB, and PMBC against *S. aureus* under idealized suspension conditions, with variables including concentration (four gradients), contact time (3, 5, 10 min), and temperature (4, 25, 37 °C). All data represent the mean of three replicate quantitative bactericidal tests. Results showed significant differences in bactericidal activity among disinfectants, with environmental factor responses displaying both commonalities and species-specific variations compared to *E. coli* (Gram-negative; detailed data in Figure 2 and Appendix Table A2).

Overall, the bactericidal efficiency ranking against *S. aureus* mirrored that against *E. coli*: CGS > PVP-I ≈ PMBC > BKB > G-DDB, but with notable species-specific differences: CGS showed extreme stability against both bacteria but achieved higher killing rates (99.99%) at lower concentrations (1:1600) within 3 min for *S. aureus* compared to *E. coli* (99.01% at 4 °C), indicating stronger adaptability to Gram-positive bacteria. PVP-I and PMBC exhibited significantly enhanced efficacy at minimal concentrations against *S. aureus* (e.g., PVP-I at 1:32 achieved 99.99% killing at 4 °C within 5 min vs. 99.91% for *E. coli*), reflecting greater sensitivity of Gram-positive bacteria to oxidizing disinfectants. BKB demonstrated faster action at medium-to-high concentrations against *S. aureus* (e.g., 1:200 achieved 100% killing at 25 °C within 3 min vs. 10 min for *E. coli*), but low concentrations remained ineffective, highlighting the most pronounced species differences among quaternary ammonium compounds. G-DDB was uniformly inefficient against both bacteria, confirming its lack of species adaptability and formulation defects independent of bacterial type.

### 3.4. Spray Disinfection Efficacy of Five Disinfectants on Stainless Steel Coupons

#### 3.4.1. Spray Disinfection Efficacy of Five Disinfectants Against *E. coli* on Stainless Steel Coupons

**Figure 3:**
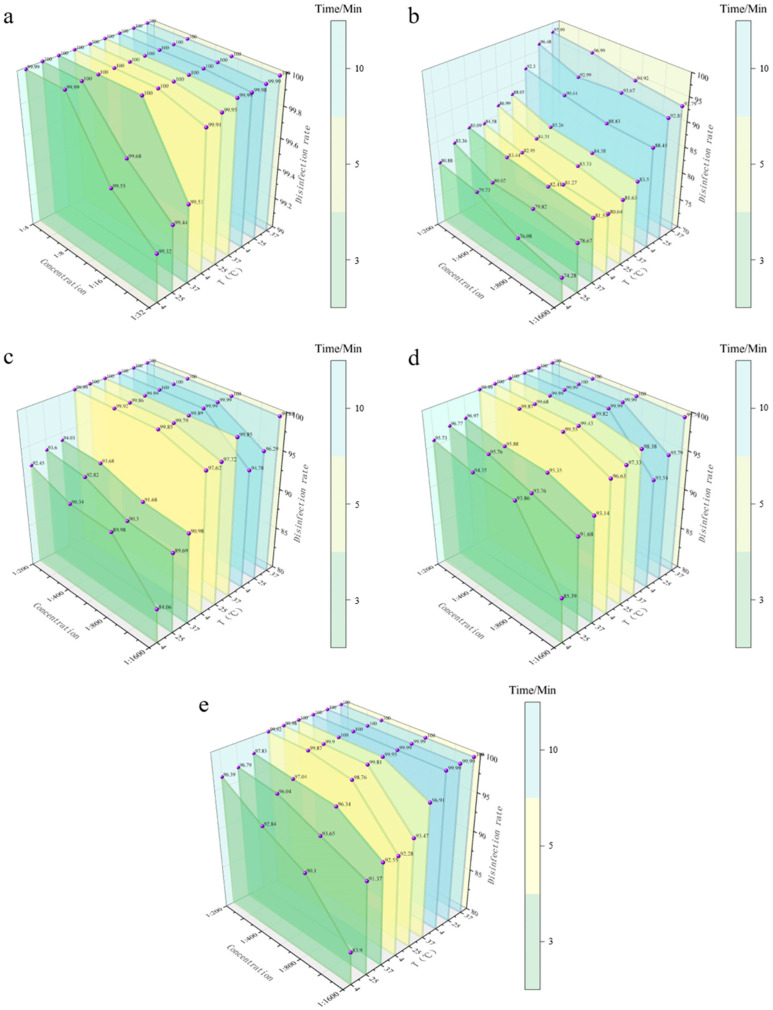
Efficacy of spray disinfection against *E. coli* on stainless steel carriers. (**a**) Bactericidal efficiency of PVP-I. (**b**) Bactericidal efficiency of BKB. (**c**) Bactericidal efficiency of CGS. (**d**) Bactericidal efficiency of G-DDB. (**e**) Bactericidal efficiency of PMBC. The *X*-axis represents disinfectant concentration, the *Y*-axis represents disinfection efficiency, and the *Z*-axis represents different temperatures. Different colors represent different contact times (3, 5, 10 min).

Stainless steel is the most commonly used hard surface material in medical devices, food processing equipment [18], and livestock facilities. *E. coli* adhering to its surface can easily cause cross-infection through contact transmission, making spray disinfection the preferred method due to its convenience and uniform coverage [19]. Using a three-factor interaction design (concentration-temperature-contact time), this study systematically evaluated the spray disinfection efficacy of PVP-I, BKB, CGS, G-DDB, and PMBC against *E. coli* on stainless steel coupons. Key objectives included quantifying killing rate variations under different conditions and revealing the dynamic mechanisms of “disinfectant adhesion-action-residue” during hard surface disinfection. All data represent the mean of three parallel spray tests. Results showed that disinfectant type determined core efficacy, with hard surface properties (e.g., smoothness, disinfectant adhesion) significantly influencing the time dependence of killing rates—distinct from suspension disinfection (rapid action) and manure bedding disinfection (high organic interference) (detailed data in Figure 3 and Appendix Table A3).

During spray disinfection on stainless steel coupons, efficacy was jointly determined by surface adhesion efficiency, active ingredient stability, and temperature adaptability: PMBC emerged as the optimal choice for its balanced performance across conditions. G-DDB demonstrated significantly improved efficacy on clean hard surfaces, overcoming its limitations in non-surface scenarios. CGS showed stable performance but required extended contact time at low concentrations/low temperatures. BKB was limited to non-urgent applications due to its reliance on prolonged adsorption. PVP-I exhibited limited efficacy due to insufficient iodine release on surfaces.

This study provides precise quantitative guidance for disinfectant selection and parameter optimization (concentration, time, temperature) in hard surface spray disinfection, particularly for stainless steel equipment in medical and food processing sectors.

#### 3.4.2. Spray Disinfection Efficacy of Five Disinfectants Against *S. aureus* on Stainless Steel Coupons

**Figure 4:**
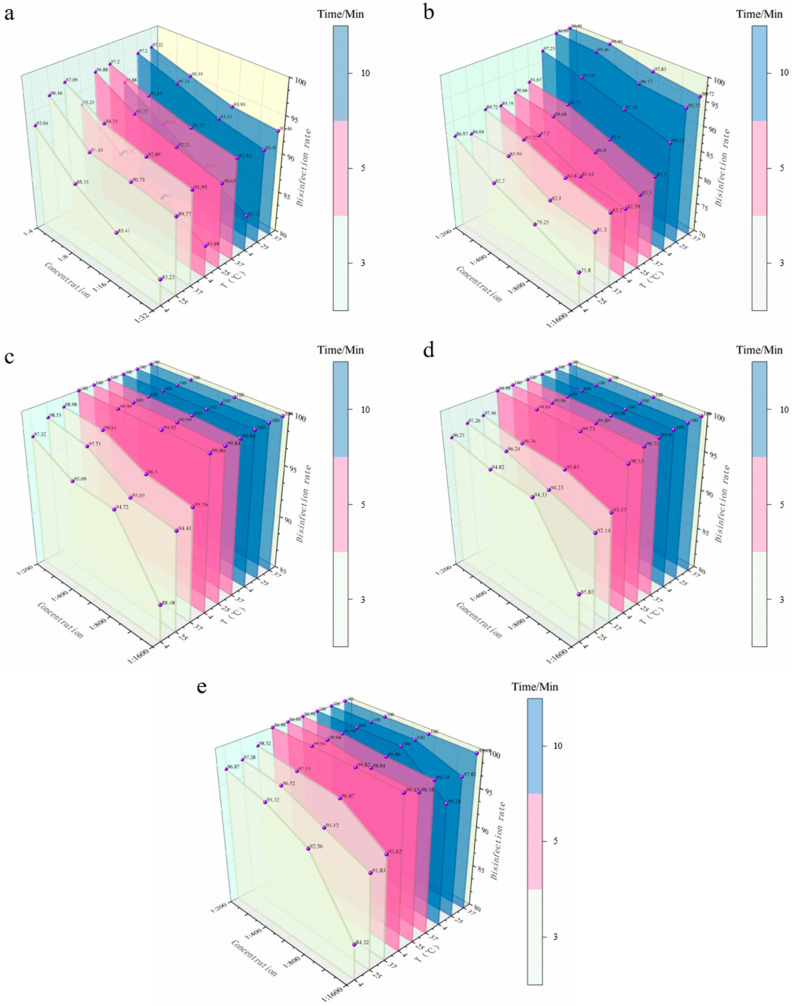
Efficacy of spray disinfection against *S. aureus* on stainless steel carriers. (**a**) Bactericidal efficiency of PVP-I. (**b**) Bactericidal efficiency of BKB. (**c**) Bactericidal efficiency of CGS. (**d**) Bactericidal efficiency of G-DDB. (**e**) Bactericidal efficiency of PMBC. The *X*-axis represents disinfectant concentration, the *Y*-axis represents disinfection efficiency, and the *Z*-axis represents different temperatures. Different colors represent different contact times (3, 5, 10 min).

*S. aureus*, a classic Gram-positive pathogen with a thick peptidoglycan layer rich in teichoic acid [20], adheres more strongly to stainless steel surfaces than Gram-negative *E. coli*, increasing cross-infection risks. Spray disinfection for hard surfaces must overcome dual challenges: bacterial adhesion and disinfectant wettability. Using the same three-factor interaction design, this study evaluated the spray disinfection efficacy of PVP-I, BKB, CGS, G-DDB, and PMBC against *S. aureus* on stainless steel coupons, focusing on species-specific efficacy differences and mechanisms of disinfectant-cell wall interactions. Results showed overall higher efficacy against *S. aureus* compared to *E. coli*, with quaternary ammonium compounds (BKB) and glutaraldehyde-based disinfectants (CGS, G-DDB) exhibiting the most pronounced species sensitivity variations (detailed data in Figure 4 and Appendix Table A4).

### 3.5. Disinfection Efficacy and Bacterial Growth–Decline Dynamics of Different Disinfectant Systems on Cow Dung Bedding

Cow dung bedding is a key site for bacterial colonization and transmission in dairy farming environments, and the dynamic changes in bacterial load directly affect the risk of bovine mastitis and other diseases [10]. This study evaluated the regulatory effects of three single disinfectants—potassium PMBC, G-DDB, and CGS—and their combinations with bleaching powder (a total of six groups) on bacteria in cow dung bedding over a 6-day monitoring period. The evaluation focused on three core dimensions: immediate disinfection efficiency, persistence, and bacterial recovery kinetics. Key metrics included the post-disinfection bacterial count and the bacterial recovery rate (reflecting the sustainability of disinfection efficacy). All data represent the mean ± standard deviation from three parallel samplings.

**Figure 5:**
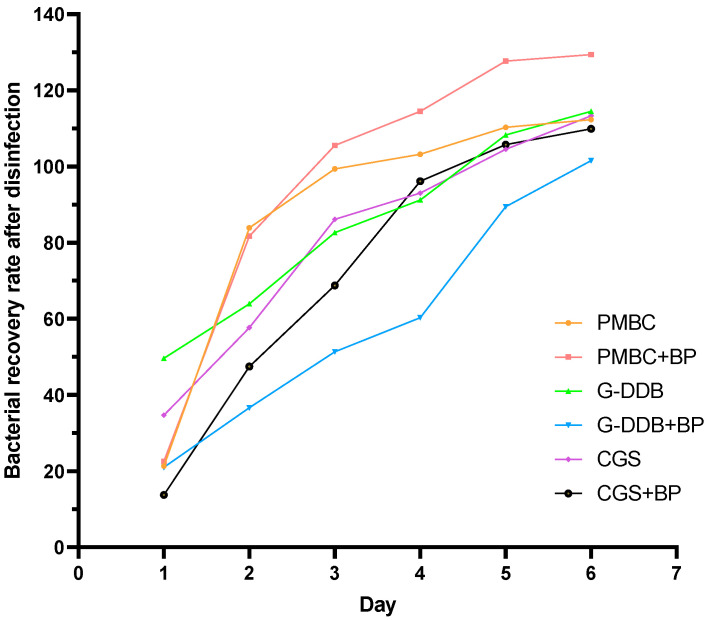
Bacterial growth-suppression dynamics and disinfection efficacy within cattle stall bedding.

Results showed that disinfectant type, combination strategy, and initial bacterial load jointly determined disinfection efficacy. The effects of combined systems were not simply “synergistic” but exhibited significant system-specific differences (see Figure 5 and Appendix Table A5 for detailed data). The combined systems showed the following characteristics: PMBC and its combination system: Limited immediate disinfection efficacy and poor persistence. G-DDB and its combination system: Initial bacterial load differences dominated the results; combination significantly enhanced efficacy, but overall performance was constrained by the low initial baseline. CGS and its combination system: Achieved the best balance between immediate efficacy and persistence, with the combination being the optimal choice.

Subsequently, based on three core metrics—immediate disinfection efficiency (1 − recovery rate), persistence days (time to first reach 100% recovery rate), and 6-day bacterial count increase ratio (Day 6 post-disinfection count/Day 1 post-disinfection count)—the six groups were ranked for overall efficacy (Table 5), further clarifying differences:

Immediate efficacy: CGS + bleaching powder (86.28%) > G-DDB + bleaching powder (79.02%) > CGS alone (65.32%). Combined systems generally outperformed single systems, but PMBC combinations showed no advantage.

Persistence: G-DDB + bleaching powder had the longest persistence (6 days), but due to its low initial baseline, practical application should consider the specific scenario; CGS + bleaching powder and CGS alone both persisted for 5 days, outperforming PMBC systems (3–4 days).

Long-term control: CGS alone had the lowest 6-day bacterial count increase ratio (3.26-fold), indicating slower bacterial regrowth despite late-stage rebound; although PMBC combination had a low increase ratio (1.67-fold), its poor initial disinfection efficacy resulted in persistently high bacterial counts.

#### Selection Strategy for Cow Dung Bedding Disinfectants

Integrating the findings of this study, for the disinfection requirements of cow dung bedding (high organic matter, high bacterial load, and the need for long-term control), the following principles should be followed:

Priority to combined systems: The CGS + bleaching powder combination achieved “high immediate efficacy (86.28% disinfection)–5-day persistence–stable without severe fluctuations” under moderate initial bacterial load, making it the optimal choice for routine bedding disinfection in large-scale dairy farms.

Alternative for low-load scenarios: The G-DDB + bleaching powder combination showed the best persistence (6 days) at low initial bacterial load (<1 × 10^6^ CFU/mL), suitable for recently cleaned bedding.

Avoid PMBC systems: Both single and combined PMBC systems exhibited “poor immediate efficacy–short persistence–pronounced rebound” and were highly susceptible to organic matter interference, making them unsuitable for cow dung bedding.

Single disinfectant as a supplement: CGS alone can serve as an alternative to combined systems (e.g., when bleaching powder storage is inconvenient), with overall efficacy still superior to other single disinfectants.

## 4. Discussion

Through a systematic experimental design, this study comprehensively evaluated the disinfection efficacy of five mainstream disinfectants—PVP-I, BKB, CGS, G-DDB, and PMBC—against *E. coli* and *S. aureus* across three typical application scenarios (bacterial suspension, stainless steel coupons, and cow dung bedding), with efficacy modulated by concentration, temperature, and contact time. The core objective was to uncover the dynamic interaction mechanisms among “microbial biological characteristics, scenario-specific environmental factors, and disinfectant action modes,” thereby providing theoretical support for precision disinfection practices in healthcare, livestock farming, and food processing, while also rectifying previous one-dimensional misconceptions regarding the efficacy of certain disinfectants.

From the perspective of species specificity, the structural differences in cell walls between *E. coli* (Gram-negative) and *S. aureus* (Gram-positive) [20] constitute the core biological basis for variations in disinfectant sensitivity. The thick peptidoglycan layer (20–80 nm) of Gram-positive bacteria, rich in teichoic acids [21], provides more specific binding sites for quaternary ammonium disinfectants (e.g., BKB). This reduces the effective concentration threshold of BKB for *S. aureus* from 1:200 (250 mg/L) (for *E. coli*) to 1:400 (125 mg/L), and a 10 min contact time at 25 °C achieved a 99.99% killing rate—significantly outperforming the 96.48% efficacy observed for *E. coli* under the same conditions. In contrast, the outer membrane of *E. coli* (containing lipopolysaccharides) forms a physical barrier [22], which can only be penetrated by disinfectants with penetration-enhancing components (e.g., non-ionic surfactants in CGS). This explains why G-DDB achieved a killing rate of only 93.54% against *E. coli* on stainless steel coupons (1:1600 concentration, 4 °C, 10 min), while reaching 100% efficacy against *S. aureus* under identical conditions. Notably, despite *S. aureus*’s stronger ability to form biofilms on stainless steel surfaces [23], its disinfection efficacy was higher—this phenomenon corrects the simplistic notion of a “positive correlation between biofilm thickness and disinfection difficulty,” suggesting that the interaction between extracellular polysaccharide components of biofilms and disinfectants may be more critical than thickness. By comparison, oxidative disinfectants such as PVP-I and PMBC exhibited the smallest species-specific differences; for instance, PMBC achieved a 100% killing rate against both bacteria at a 1:800 concentration with a 10 min contact time. This is attributed to the free radicals (OH^−^, SO_4_^−^) released by PMBC, which damage cell wall components through non-specific oxidative reactions [24] without relying on species-specific binding—consistent with existing findings on the broad-spectrum activity of oxidative disinfectants.

The regulation of disinfection efficacy by scenario-specific environmental factors exhibited distinct hierarchical characteristics: interference from organic matter (in the cow dung bedding scenario) was the most prominent, followed by adhesion limitations on hard surfaces (in the stainless steel coupon scenario), while the suspension scenario—devoid of additional interference—served as a benchmark for intrinsic disinfectant efficacy. In the high-organic-matter environment of cow dung bedding, components such as proteins and polysaccharides undergo non-specific binding with disinfectants [25], significantly reducing their effective concentration. For the PMBC alone group (1:200 concentration), the bacterial recovery rate reached 112.3% after 6 days, failing to maintain long-term inhibitory effects. However, when G-DDB was combined with bleaching powder, hypochlorous acid preferentially reacted with organic matter to protect glutaraldehyde from quenching, resulting in a 6-day recovery rate of only 101.57% and a bacterial load consistently maintained at the 10^5^ CFU/mL level (most other groups reached 10^6^ CFU/mL). These results confirm that high-organic-matter scenarios require prioritizing “anti-quenching” or “synergistic combination” disinfectant systems, rather than simply relying on high concentrations of single-component disinfectants. For hard surfaces like stainless steel coupons, low disinfectant adhesion (due to surface smoothness, with a post-spray droplet contact angle of approximately 60°—30% higher than that in *E. coli* suspension) and rapid local concentration decline caused by evaporation emerged as key limiting factors. For PVP-I, the easy evaporation loss of iodide ions [26] prevented it from achieving a 100% killing rate across all conditions; even at a 1:4 concentration (37 °C, 10 min) on stainless steel, its efficacy only reached 97.32%. In contrast, PMBC maintained high free radical stability on surfaces [27], achieving a 99.99% killing rate at a 1:1600 concentration with a 10 min contact time. For BKB, surface adsorption equilibrium required 10 min, delaying its efficacy inflection point by 5 min compared to the suspension scenario—further confirming that surface adsorption acts as a rate-limiting step for quaternary ammonium disinfectants. As an ideal benchmark, the bacterial suspension scenario clearly reflected the theoretical optimal efficacy of disinfectants: CGS achieved a 100% killing rate at a 1:1600 concentration (37 °C, 5 min), approaching “theoretical bactericidal efficiency,” while G-DDB required a higher concentration (1:800) to achieve the same effect under identical conditions—revealing its inherent limitation of lacking synergistic enhancement in single-component formulations.

Based on the above findings, combined with the high density, high pollution risk, and core facility characteristics of large-scale dairy farms, a targeted scenario-specific disinfectant selection strategy can be developed:

For high-risk facilities in large-scale dairy farms (e.g., stainless steel teat cup clusters of milking machines, stainless steel nursery pens in calf barns, and veterinary treatment tables), CGS or PMBC should be prioritized. These facilities are directly associated with the transmission of critical diseases such as bovine mastitis and calf diarrhea. The high efficacy and broad temperature adaptability (stable at 4–37 °C) of these two disinfectants meet the needs of dairy farms, which face large daily temperature fluctuations and high disinfection frequency—making them particularly suitable for immediate disinfection before and after milking.

For core pollution scenarios in large-scale dairy farms (e.g., high-density cow dung cubicle bedding), the CGS + bleaching powder combination is optimal. Under conditions of an initial bacterial load of 4.09 × 10^6^ CFU/mL (close to the daily pollution level of dairy farm bedding), the 6-day bacterial recovery rate of this combination was only 109.92%—far lower than that of the PMBC alone group (112.3%) and G-DDB alone group (114.48%). It can effectively control the risk of hoof diseases and mastitis caused by bacterial growth in bedding, adapting to the regular disinfection process of large-scale dairy farm bedding (e.g., once every 3 days).

For feed processing and transportation scenarios in dairy farms (e.g., stainless steel inner walls of feed mixers, stainless steel components of TMR delivery pipelines), BKB or G-DDB can be used. These scenarios require low disinfection frequency (e.g., once a day); the cost advantages and medium-temperature stability of the two disinfectants balance efficacy and breeding costs, with no significant damage to feed components.

For low-temperature storage scenarios in large-scale dairy farms (e.g., cold-chain milk tanks, stainless steel containers for silage transfer), PMBC or CGS is recommended, while low-concentration BKB, which is sensitive to low temperatures, should be avoided. These scenarios are directly related to raw milk safety and silage quality [28], and the low-temperature efficacy of the disinfectants can reduce the risk of bacterial contamination in the cold chain.

Meanwhile, the research design of this study for disinfection in large-scale dairy farms still has three limitations, which need to be improved in future work based on the actual needs of dairy farms:

First, although the *E. coli* and *S. aureus* strains used in this study were core pollution strains isolated and identified from large-scale dairy farm environments (e.g., bedding manure, inner walls of milking machine pipelines, and calf barn floors)—which can reflect the characteristics of real pollution bacteria in dairy farms—they do not cover multidrug-resistant strains that may exist in dairy farms (e.g., bovine-derived *E. coli* carrying the blaCTX-M resistance gene [29], bovine-derived *S. aureus* carrying the mecA gene [30]). Such resistant strains are prone to enrichment in large-scale dairy farms due to long-term use of antibiotics, and their sensitivity to disinfectants may be lower [31]. Additionally, the study did not evaluate the efficacy of disinfectants against specific conditional pathogens in dairy farms (e.g., *Mycoplasma bovis*, *Streptococcus agalactiae*).

Second, there is a lack of verification of molecular mechanisms for strains isolated from dairy farms. Without combining the genomic characteristics of dairy farm strains to analyze the impact of disinfectants on the unique metabolic pathways of dairy farm strains through transcriptomics, proteomics, or single-cell sequencing technology, it is difficult to explain in depth the efficacy differences in disinfectants in specific polluted environments of dairy farms (e.g., equipment surfaces containing milk residues and feed fibers) at the molecular level.

Third, the residual risk of disinfectants in practical applications in large-scale dairy farms was not evaluated; only immediate and short-term (6-day) disinfection efficacy was focused on. However, large-scale dairy farms use large amounts of disinfectants (e.g., a single bedding disinfection covers thousands of square meters), and post-disinfection residues easily enter the pasture’s return-to-field system with manure, which may have potential inhibitory effects on soil nitrogen-fixing microorganisms [32] and dairy cow rumen probiotics. At the same time, discharge with cleaning wastewater may affect the microbial community of water bodies around the pasture [33]. Therefore, a multi-objective evaluation system integrating “disinfection efficacy, dairy cow health, and pasture ecological cycle” needs to be established.

Future research can focus on the pain points of large-scale dairy farms, such as developing nano-carrier compound disinfectants suitable for the CIP (Clean-in-Place) system of dairy farms, and optimizing formulations based on the genetic characteristics of drug-resistant strains isolated from dairy farms. At the same time, combined with the disinfection process of large-scale dairy farms, the stability and residual safety of disinfectants under high-frequency use should be verified, promoting the development of disinfectants toward adapting to large-scale dairy farms and balancing disease prevention and control with ecological safety.

## 5. Conclusions

This study systematically evaluated the bactericidal efficacy of five disinfectants against two key pathogenic bacteria in dairy farms across different scenarios, and revealed the complex interaction patterns among disinfectant type, environmental conditions, and bacterial characteristics.

Results showed that CGS exhibited the broadest adaptability and stability, with excellent performance across scenarios including bacterial suspension, stainless steel surfaces, and cow dung bedding. PMBC, owing to the high stability of its free radicals, emerged as an ideal choice for low-temperature environments. PVP-I demonstrated high efficacy but was limited by surface conditions. BKB required longer contact time and was temperature-sensitive. G-DDB exhibited limited efficacy in high-organic-matter environments.

This study provides precision scenario-specific disinfection strategies for dairy farms, and emphasizes the notable advantages of combined systems (e.g., CGS + BP) in controlling bacterial rebound in high-pollution areas. These findings not only improve biosafety management in dairy farms but also offer valuable references for disinfection practices in fields such as healthcare and food processing. Meanwhile, the study highlights the need for future research on drug-resistant strains, molecular mechanisms, and ecological impacts.

## Figures and Tables

**Table 1:** The selection of neutralizers for five commonly used disinfectants.

Disinfectant Abbreviation	Main Component(s)	Dilution Ratio (mg/L)	Neutralizer
PVP-I	5% Iodine	156.25~1250	0.1% Sodium Thiosulfate Solution
BKB	5% Benzalkonium Bromide	31.25~250	3% Tween 80
CGS	15% Glutaraldehyde + 10% Benzalkonium Chloride	62.5~500	3% Tween-80 + 0.5% Lecithin + 0.3% Glycine
G-DDB	5% Glutaraldehyde + 5% Didecyldimethylammonium Bromide	62.5~500	3% Tween-80 + 0.5% Lecithin + 0.3% Glycine
PMBC	Available Chlorine ≥ 10%	62.5~500	2% Tween-80 + 0.1% Lecithin + 0.5% Sodium Thiosulfate

**Table 2:** Results of Neutralizer Identification Test.

Disinfectant Abbreviation	*E. coli*/(10^7^ CFU/mL)						Error Rate (%)
	Group 1	Group 2	Group 3	Group 4	Group 5	Group 6	
PVP-I	0	8	1.66	1.87	1.70	0	4.84
BKB	0	9	1.70	1.73	1.68	0	1.04
CGS	0	9	1.32	1.21	1.67	0	12.86
G-DDB	0	11	1.24	1.08	1.40	0	8.60
PMBC	0	8	1.54	1.87	1.56	0	8.58

**Table 3:** MIC Values of the Five Disinfectants Against the Two Bacterial Strains (dilution ratio).

Pathogenic Microorganism	MIC Value (Dilution Ratio) of Disinfectants				
	PVP-I	PMBC	BKB	CGS	G-DDB
*E. coli*	1:8	1:800	1:1280	1:3840	1:2400
*S. aureus*	1:8	1:800	1:1280	1:3840	1:2400

**Table 4:** MBC Values of the Five Disinfectants Against the Two Bacterial Strains (dilution ratio).

Pathogenic Microorganism	MIC Value (Dilution Ratio) of Disinfectants				
	PVP-I	PMBC	BKB	CGS	G-DDB
*E. coli*	1:4	1:400	1:160	1:640	1:480
*S. aureus*	1:4	1:400	1:160	1:640	1:480

**Table 5:** Bacterial Growth–Decline Dynamics and Disinfection Efficacy on Cow Dung Bedding.

Group	Immediate Disinfection Efficiency (%)	Persistence Days (days)	6-Day Bacterial Count Increase Ratio	Overall Efficacy Rank
CGS + BP	86.28	5	7.98	1
G-DDB + BP	79.02	6	4.83	2
CGS alone	65.32	5	3.26	3
G-DDB alone	50.36	5	2.27	4
PMBC alone	21.30	4	1.43	5
PMBC + BP	22.60	3	1.67	6

## Data Availability

The original contributions presented in this study are included in the article. Further inquiries can be directed to the corresponding author.

## Abbreviations

The following abbreviations are used in this manuscript:

PVP-I	Polyvinylpyrrolidone–Iodine Solution
BKB	Benzalkonium Bromide Preparation
CGS	Compound Glutaraldehyde Preparation
G–DDB	Glutaraldehyde–Didecyldimethylammonium Bromide Solution
PMBC	Potassium Peroxymonosulfate Complex Powder
BP	Bleaching Powder

## Appendix A

**Table A1:** Bacterial reduction rate of disinfectants against *E. coli* in aqueous suspension.

Disinfectants	3 min (%)	5 min (%)	10 min (%)	3 min (%)	5 min (%)	10 min (%)	3 min (%)	5 min (%)	10 min (%)
	4 °C	25 °C	37 °C	4 °C	25 °C	37 °C	4 °C	25 °C	37 °C
PVP-I									
1:4	99.99	100.00	100.00	100.00	100.00	100.00	100.00	100.00	100.00
1:8	99.99	100.00	100.00	100.00	100.00	100.00	100.00	100.00	100.00
1:16	99.55	99.68	100.00	100.00	100.00	100.00	100.00	100.00	100.00
1:32	99.32	99.44	99.51	99.91	99.95	99.99	99.98	99.99	100.00
BKB									
1:200	97.23	96.30	99.99	99.99	99.20	100.00	100.00	100.00	100.00
1:400	92.50	96.20	98.18	95.23	98.30	99.99	99.99	100.00	100.00
1:800	90.63	91.68	93.45	92.46	95.42	97.19	95.73	100.00	100.00
1:1600	84.62	87.25	92.18	88.21	90.26	95.80	93.25	96.20	98.30
CGS									
1:200	100.00	100.00	100.00	100.00	100.00	100.00	100.00	100.00	100.00
1:400	99.99	100.00	100.00	100.00	100.00	100.00	100.00	100.00	100.00
1:800	99.69	100.00	100.00	99.99	100.00	100.00	100.00	100.00	100.00
1:1600	99.01	96.40	98.20	99.20	100.00	100.00	99.87	100.00	100.00
G-DDB									
1:200	96.59	78.32	98.44	80.73	99.16	99.20	85.54	100.00	100.00
1:400	86.78	74.05	85.58	75.11	91.71	95.85	81.08	98.40	99.99
1:800	58.68	73.65	73.83	74.91	76.89	78.48	79.56	88.38	81.43
1:1600	54.89	70.46	72.65	65.80	70.15	75.51	75.75	60.92	80.72
PMBC									
1:200	100.00	100.00	100.00	100.00	100.00	100.00	100.00	100.00	100.00
1:400	93.10	98.33	98.74	100.00	100.00	100.00	100.00	100.00	100.00
1:800	91.54	95.46	97.80	99.62	100.00	100.00	100.00	100.00	100.00
1:1600	88.91	95.00	95.60	94.70	95.00	97.30	100.00	100.00	100.00

**Table A2:** Bacterial reduction rate of disinfectants against *S. aureus* in aqueous suspension.

Disinfectants	3 min (%)	5 min (%)	10 min (%)	3 min (%)	5 min (%)	10 min (%)	3 min (%)	5 min (%)	10 min (%)
	4 °C	25 °C	37 °C	4 °C	25 °C	37 °C	4 °C	25 °C	37 °C
PVP-I									
1:4	100.00	100.00	100.00	100.00	100.00	100.00	100.00	100.00	100.00
1:8	100.00	100.00	100.00	100.00	100.00	100.00	100.00	100.00	100.00
1:16	100.00	100.00	100.00	100.00	100.00	100.00	100.00	100.00	100.00
1:32	99.84	99.98	100.00	99.99	100.00	100.00	100.00	100.00	100.00
BKB									
1:200	97.93	100.00	99.99	99.99	100.00	100.00	100.00	100.00	100.00
1:400	96.82	96.21	99.89	99.99	99.99	100.00	100.00	100.00	100.00
1:800	91.25	92.10	94.80	93.61	96.80	97.90	100.00	100.00	100.00
1:1600	85.80	88.30	93.20	92.29	96.30	97.20	93.72	98.21	99.25
CGS									
1:200	100.00	100.00	100.00	100.00	100.00	100.00	100.00	100.00	100.00
1:400	100.00	100.00	100.00	100.00	100.00	100.00	100.00	100.00	100.00
1:800	100.00	100.00	100.00	100.00	100.00	100.00	100.00	100.00	100.00
1:1600	99.99	99.99	99.99	99.99	100.00	100.00	100.00	100.00	100.00
G-DDB									
1:200	97.37	78.95	99.23	81.38	99.96	100.00	86.23	100.00	100.00
1:400	88.01	75.10	86.80	76.72	93.68	97.91	81.90	99.39	100.00
1:800	59.51	74.70	74.88	76.52	78.54	80.16	80.36	89.27	81.58
1:1600	55.67	71.46	73.68	67.21	71.65	77.13	76.52	61.54	77.33
PMBC									
1:200	100.00	100.00	100.00	100.00	100.00	100.00	100.00	100.00	100.00
1:400	100.00	100.00	100.00	100.00	100.00	100.00	100.00	100.00	100.00
1:800	97.75	100.00	100.00	100.00	100.00	100.00	100.00	100.00	100.00
1:1600	93.42	95.40	97.67	100.00	100.00	100.00	100.00	100.00	100.00

**Table A3:** Efficacy of spray disinfection against *E. coli* on stainless steel carriers.

Disinfectants	3 min (%)	5 min (%)	10 min (%)	3 min (%)	5 min (%)	10 min (%)	3 min (%)	5 min (%)	10 min (%)
	4 °C	25 °C	37 °C	4 °C	25 °C	37 °C	4 °C	25 °C	37 °C
PVP-I									
1:4	92.56	94.27	96.16	92.81	94.92	96.84	93.55	95.30	97.03
1:8	87.28	89.26	93.77	88.13	92.19	94.21	89.92	94.83	94.80
1:16	84.92	86.93	90.13	86.09	91.82	91.51	87.03	92.10	92.87
1:32	81.37	84.55	86.07	82.36	87.19	90.99	83.79	89.02	92.84
BKB									
1:200	80.88	83.36	85.09	84.58	86.99	88.05	92.30	96.48	97.99
1:400	79.73	80.02	83.44	82.95	84.31	85.26	90.44	92.99	96.99
1:800	76.08	79.82	82.41	81.27	83.33	84.38	88.83	93.67	94.92
1:1600	74.28	78.67	81.54	80.64	81.63	83.50	88.45	92.81	93.79
CGS									
1:200	92.45	93.60	94.03	99.99	100.00	100.00	100.00	100.00	100.00
1:400	90.34	92.82	93.68	99.92	99.86	99.99	100.00	100.00	100.00
1:800	89.98	90.30	91.68	99.85	99.79	99.89	99.99	99.99	100.00
1:1600	84.06	89.69	90.98	97.62	97.72	99.85	94.78	96.29	99.88
G-DDB									
1:200	95.73	96.77	96.97	99.99	100.00	100.00	100.00	100.00	100.00
1:400	94.35	95.76	95.88	99.87	99.68	99.99	99.99	100.00	100.00
1:800	93.86	93.76	95.35	99.55	99.43	99.82	99.99	99.99	100.00
1:1600	85.39	91.68	93.14	96.63	97.33	98.38	93.54	95.79	99.76
PMBC									
1:200	96.39	96.79	97.83	99.92	99.98	100.00	100.00	100.00	100.00
1:400	92.84	96.04	97.04	99.87	99.90	100.00	100.00	100.00	100.00
1:800	90.10	93.65	96.34	98.76	99.81	99.95	99.99	99.99	100.00
1:1600	83.90	91.37	92.55	92.28	93.47	96.91	99.99	99.99	100.00

**Table A4:** Efficacy of spray disinfection against *S. aureus* on stainless steel carriers.

Disinfectants	3 min (%)	5 min (%)	10 min (%)	3 min (%)	5 min (%)	10 min (%)	3 min (%)	5 min (%)	10 min (%)
	4 °C	25 °C	37 °C	4 °C	25 °C	37 °C	4 °C	25 °C	37 °C
PVP-I									
1:4	93.04	96.16	97.09	93.25	96.88	97.20	93.88	97.20	97.32
1:8	88.33	91.45	94.25	89.37	93.77	95.33	90.65	95.33	95.55
1:16	85.41	90.71	92.89	86.70	92.21	93.77	87.54	93.15	93.93
1:32	83.23	89.77	91.95	83.88	90.65	92.83	84.42	91.90	93.46
BKB									
1:200	86.97	86.04	89.72	89.19	90.66	91.67	97.25	99.99	99.99
1:400	82.20	85.94	87.72	87.20	89.68	90.77	95.16	99.46	99.99
1:800	79.25	82.10	84.80	83.61	86.80	87.90	92.53	96.53	97.83
1:1600	75.80	81.30	83.20	82.29	83.30	85.20	90.25	95.72	96.72
CGS									
1:200	97.32	98.53	98.98	100.00	100.00	100.00	100.00	100.00	100.00
1:400	95.09	97.71	98.61	99.99	100.00	100.00	100.00	100.00	100.00
1:800	94.72	95.05	96.50	99.92	99.99	100.00	100.00	100.00	100.00
1:1600	88.48	94.41	95.76	99.86	99.84	99.84	100.00	100.00	100.00
G-DDB									
1:200	96.21	97.26	97.46	99.99	100.00	100.00	100.00	100.00	100.00
1:400	94.82	96.24	96.36	99.91	99.96	100.00	100.00	100.00	100.00
1:800	94.33	94.23	95.83	99.73	99.89	99.98	100.00	100.00	100.00
1:1600	85.82	92.14	93.57	98.53	99.71	99.90	100.00	100.00	100.00
PMBC									
1:200	96.87	97.28	98.32	99.99	99.99	99.99	100.00	100.00	100.00
1:400	95.32	96.52	97.53	99.94	99.94	99.99	100.00	100.00	100.00
1:800	92.56	94.12	96.82	99.82	98.84	99.46	100.00	100.00	100.00
1:1600	84.32	91.83	93.02	99.47	98.58	99.19	95.28	97.82	99.98

**Table A5:** Bacterial growth-suppression dynamics and disinfection efficacy within cattle stall bedding.

Group	Item	Day 1	Day 2	Day 3	Day 4	Day 5	Day 6
PMBC	Bacterial Count Before Disinfection (10^6^ cfu/mL)	1.55 ± 0.82	-	-	-	-	-
	Bacterial Count After Disinfection (10^6^ cfu/mL)	1.22 ± 0.17	1.30 ± 0.73	1.54 ± 1.21	1.60 ± 0.73	1.71 ± 1.70	1.74 ± 1.17
	Bacterial Recovery Rate (%)	21.3	83.9	99.4	103.2	110.3	112.3
PMBC + BP	Bacterial Count Before Disinfection (10^6^ cfu/mL)	2.35 ± 0.17	-	-	-	-	-
	Bacterial Count After Disinfection (10^6^ cfu/mL)	1.82 ± 0.17	1.92 ± 2.56	2.48 ± 1.70	2.69 ± 2.23	3.00 ± 1.59	3.04 ± 1.82
	Bacterial Recovery Rate (%)	22.6	81.7	105.5	114.5	127.7	129.4
G-DDB	Bacterial Count Before Disinfection (10^6^ cfu/mL)	3.97 ± 1.25	-	-	-	-	-
	Bacterial Count After Disinfection (10^6^ cfu/mL)	2.00 ± 1.03	2.54 ± 1.23	3.28 ± 0.83	3.62 ± 0.96	4.30 ± 1.03	4.54 ± 2.83
	Bacterial Recovery Rate (%)	49.64	63.93	82.62	91.23	108.31	114.48
G-DDB + BP	Bacterial Count Before Disinfection (10^5^ cfu/mL)	9.50 ± 1.01	-	-	-	-	-
	Bacterial Count After Disinfection (10^5^ cfu/mL)	2.00 ± 1.06	3.48 ± 2.74	4.87 ± 2.96	5.74 ± 0.94	8.51 ± 1.26	9.66 ± 2.31
	Bacterial Recovery Rate (%)	20.98	36.62	51.34	60.32	89.43	101.57
CGS	Bacterial Count Before Disinfection (10^6^ cfu/mL)	5.56 ± 0.71	-	-	-	-	-
	Bacterial Count After Disinfection (10^6^ cfu/mL)	1.93 ± 1.09	3.21 ± 0.31	4.79 ± 0.76	5.22 ± 2.29	5.81 ± 0.74	6.29 ± 1.12
	Bacterial Recovery Rate (%)	34.68	57.72	86.13	93.02	104.54	113.33
CGS + BP	Bacterial Count Before Disinfection (10^6^ cfu/mL)	4.09 ± 1.14	-	-	-	-	-
	Bacterial Count After Disinfection (10^6^ cfu/mL)	0.56 ± 0.34	1.94 ± 0.98	2.81 ± 1.43	3.93 ± 2.00	4.32 ± 2.20	4.49 ± 2.28
	Bacterial Recovery Rate (%)	13.72	47.47	68.73	96.12	105.74	109.92
